# Effects of strip cropping with reducing row spacing and super absorbent polymer on yield and water productivity of oat (*Avena sativa L.*) under drip irrigation in Inner Mongolia, China

**DOI:** 10.1038/s41598-022-15418-w

**Published:** 2022-07-06

**Authors:** Lu Tian, Jing-hui Liu, Sheng Zhang, Bao-ping Zhao, Jun-zhen Mi, Ying-hao Li, Feng-wu Wang

**Affiliations:** 1 College of Agronomy, Inner Mongolia Agricultural University, No.275, Xin Jian East Street, Hohhot, 010019 China; 2 Agriculture and Forestry Sciences of Ulanqab, Jining, 012000 China

**Keywords:** Agroecology, Agroecology

## Abstract

With the serious shortage of water resources and the development of water-saving agriculture, the application of drip irrigation has been paid more and more attention. But there was lack of oat planting methods suitable for drip irrigation, currently. In order to establish an efficient oat planting method for drip irrigation, a study was conducted at Agriculture and Forestry Sciences of Ulanqab, Inner Mongolia during the season (2019–2020) to evaluate the effect of strip cropping with reducing row spacing and super absorbent polymer on the yield and water use efficiency of oat. To conduct the field trials, a split plot system in three replications was established. Three planting patterns were in the main plots, including conventional cropping with 20 cm equal row spacing (PA), strip cropping with the 15 cm row spacing (PB) and strip cropping with the 10 cm row spacing (PC), and two super absorbent polymer levels were in the subplots, including 22.5 kg ha^−2^ (Y) and 0 (N). The results showed that, compared with PA, PB and PC both decreased the irrigation volumes by 4.5–18.4 mm, and the irrigation volumes of PB was lower than that of PC. When super absorbent polymers were applied, compared with PA, PB significantly increased grain yield and above-ground biomass, but PC had the opposite effects. The grain yield and above-ground biomass of PB significantly increased by 16.65% and 7.31% on average in two years, respectively. And the increasing of grain yield was attributed by the significant increasing of pike number and kernel number per spike. But when super absorbent polymers were not applied, PB had no significant effects on grain yield and above-ground biomass. PB also had the significant effects on regulating water use of oats weather or not super absorbent polymers were applied, it significantly increased the precipitation ratio by 2.64% (PBY) and 2.13% (PBN) and decreased irrigation ration by 3.32% (PBY) and 5.28% (PBN) on average in two years. Although PB and PC both decreased the total evapotranspiration, but PB increased WUE and PC deceased WUE. The WUE of PB increased by 19.70% (PBY) and 9.87% (PBN) on average in two years. Also PB had the highest economic benefits in all treatments**.** In conclusion, a drip irrigation oat planting pattern was proposed, which the row spacing is 15 cm, adjusted the equal row spacing planting to 8-row strip planting, with a belt spacing of 30 cm, combined with the application of 22.5 kg ha^−2^ applying super absorbent polymers. And this oat planting pattern is a viable strategy to improve oat productivity.

## Introduction

Oat (*Avena sativa L*.) is a small-grain cereal crop and produced on a global scale, it is one of the eight major food crops in the world, and its total output ranks fifth^1^. Oats have a long history of use as a beneficial health food, especially the grain is rich in β- glucan^2^. China is the birthplace of naked oats with the largest planting area in the world, also Inner Mongolia is the largest oat producing area in China which accounts for more than 35% of the total area of the country^3^. And the unique geographical environment and location advantages determine the oats in Inner Mongolia have the high β- glucan content and quality. With the increasing demand for oats, according estimated consumption and planting area, the total output of oat need to 1.7 million tons in Inner Mongolia which can meet the edible needs in China. Above all, the development of oat industry in Inner Mongolia has great potential and space.

Due to the increase in population worldwide and global climate change, the demands for food have been increased and the pressure on water resources is increasing^4,5^. Currently, the situation that the majority of global water resources are used for agricultural production, especially for irrigation which accounts for 67% to 70% of current global water withdrawal^6,7^ and is as high as 90% in some countries, such as India^8,9^, has resulted into more and more researchers worldwide to focus on the means to produce more food with minimum water consumption^10–12^. There are many strategies, such as drip irrigation, super absorbent polymers, mulches, and conversation tillage, used to reduce water consumption and improve crop water use efficiency in the arid and semi-arid regions^13–15^.

Over the past few decades, droughts and reduction of water resources in arid and semi-arid areas has resulted into the modification of agricultural irrigation systems. And it is particularly important to alter surface irrigation technology to techniques to modern methods of irrigation to improve water productivity^16^. The development of drip irrigation technology enriches the agricultural measures of water-saving irrigation. It can directly supply water to crops. By adjusting water supply, the regulation of water and fertilizer can be realized, which can promote the growth of crops. Currently, drip irrigation has been widely used, especially for wide row crops. There are also some reports on the research of dense planting crops, especially in the cultivation and application of wheat^17–19^ investigated that, compared with the basin irrigation method, although the drip irrigation reduced wheat grain yield by 10.8%, but it increased water use efficiency by 24.24% by reducing water-usage. And there were different effects of tape spacing on wheat in drip irrigation. Yan^20^ indicated that the highest level of spring wheat yield was 8964 kg ha^−1^ at 60 cm tape spacing. Chen^21^ indicated that wheat yield decreased with the increasing of tape spacing from 60 to 90 cm in drip irrigation, and Shock^22^ also showed a similar results that when the tape spacing in drip irrigation is higher than 65 cm, the kernel and biomass yield of wheat decrease caused by the required water requirements not being met. Above all, maybe the better tape spacing on dense planting crops in drip irrigation is 60 cm, such as wheat.

Application of appropriate super absorbent polymers (SAPs) to improve crop water use efficiency has become an increasingly popular option to improve the sustainability of dryland agriculture in arid and semi-arid areas. SAPs have the effects of absorbing and retaining water due to their hydrophilic nature. The water absorbed by SAPs can reach 400–600 times of its dry weight, when they are incorporated into soil, they retain large quantities of water which can be released as required by the plant, so it can be used to increase the soil water retention^23^. Several researches showed that SAPs not only have effects on promoting plant growth by increasing the plant-available water in soil, but also prolonging the survival of plants under condition of water shortage^24^. Also, SAPs can increased crop yield and water use efficiency^25–29^. And there were similar results showed on oats, SAPs can promote plants growth, dry matter accumulation and yield formation of oat, which the grain yield increased by 10.80%-86.30%.

One of effective strategies of improving water management is the regulation of water holding properties of soil to optimize crop water needs. And a combination of more efficient water management methods and technologies can has better effects on reducing the consumption of water in irrigation agriculture. Recent years, with the mature development of drip irrigation, under the comprehensive cultivation goal of water saving, water conservation and high efficiency, there were many studies on the application of super absorbent polymers in drip irrigation. The results show that the application of super absorbent polymers under drip irrigation can better promote crop growth, improve water use efficiency and save water consumption^30–32^.

Generally, oat has been grown on barren land and has been considered as one of low input crops in the world^33^, and these are the same in Inner Mongolia, China. Currently, oat planting technology is single and backward and lack of the cultivation techniques for high yield, especially under irrigation conditions. There are few researches of drip irrigation on oats, several studies showed drip irrigation had obviously water saving effects compared with surface irrigation^34^, and the better irrigation volume was 120 mm^35^. Compared with no application of super absorbent polymers in drip irrigation, super absorbent polymers increased oat grain yield by 2.95%-12.14%^35^. And to establish an oat planting method suitable for irrigation conditions is particularly important in improving oat productivity.

In the past studies of drip irrigation in dense planting crops cultivations, initially the prevalent cropping layout is based on surface irrigation methods, while when the method of irrigation was changed, the cropping layout must also be changed in order to optimally utilize the existing potentials inherent in modern irrigation systems. At the same time, the results obtained in specific studies such as those by Yan^20^, Chen^21^ and Shock^22^ indicate that 60 cm maybe the better tape spacing on dense planting crops in drip irrigation. Thus, the focal question underlying the present study is to whether the increased yield and water productivity in oat, could be achieved by changing the cropping layout of oat rows in between the drip irrigation tubes which is 60 cm and applying super absorbent polymers.

The present study was conducted on a field-scale to investigate the effects of strip cropping patterns and super absorbent polymers under dirp irrigation on the yield and water productivity index of oat crops, as well as a comparison with the conventional cropping with 20 cm equal row spacing which is the prevailing planting method in the region. And this paper analyzed whether the strip planting mode of drip irrigation had the effects of water saving and high efficiency, and then selected a suitable planting mode for oats under drip irrigation.

## Materials and methods

### Site description

A two-year field experiment was conducted at Agriculture and Forestry Sciences of Ulanqab in Inner Mongolia (40.9232°N, 113.1196°E). This region was a typical temperate continental monsoon climate with large variation in rainfall quantity and distribution. It has the elevation of about 1962 m, with annual rainfall of 376 mm and mean annual temperature of 4.5 ^◦^C, respectively. The rainfall mainly was unevenly distributed and concentrated in July and August. The soil type was classified as chestnut soil, the descriptive baseline soil properties were measured according to standard methods^36,37^ and are shown in Table 1.

**Table 1:** The chemical properties of soil in the experimental site of 0-20 cm.

Soil chemical properties index	Value	
	Year 2019	Year 2020
Organic matter(g·kg^−1^)	18.21	17.56
Total nitrogen(g·kg^−1^)	0.71	0.68
Total potassium(g·kg^−1^)	16.31	16.54
Total phosphorus(g·kg^−1^)	0.46	0.50
Alkali hydrolyzed nitrogen(mg·kg^−1^)	111.07	110.89
Available potassium(mg·kg^−1^)	153.01	158.23
Available phosphorus(mg·kg^−1^)	9.23	9.54
pH value	7.4	7.6
EC (µs/cm)	198	247

**Figure 1:**
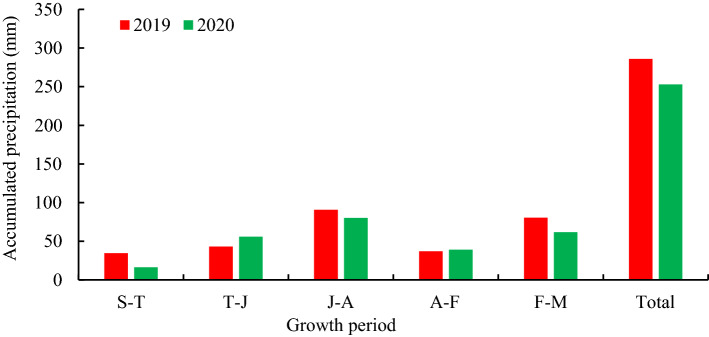
The accumulated precipitation at the different growth period. *Note* S-T, from Sowing to Tillering; T-J, from Tillering to Jointing; J-A, from Jointing to Anthesis; A-F, from Anthesis to Filling; F-M, from Filling to Maturity.

Precipitation during the growth period of oat was shown in Fig. 1. The total precipitation was 285.8 mm (2019), 252.9 mm (2020) respectively during the whole growing period of oat at the experimental station. And the precipitation showed the same trend during the whole growing period of oat in two years, both showed lower in early reproductive period, after jointing showed increased and reached the highest in blooming, after blooming showed decreased and increased after filling.

### Experimental design and field management

**Figure 2:**
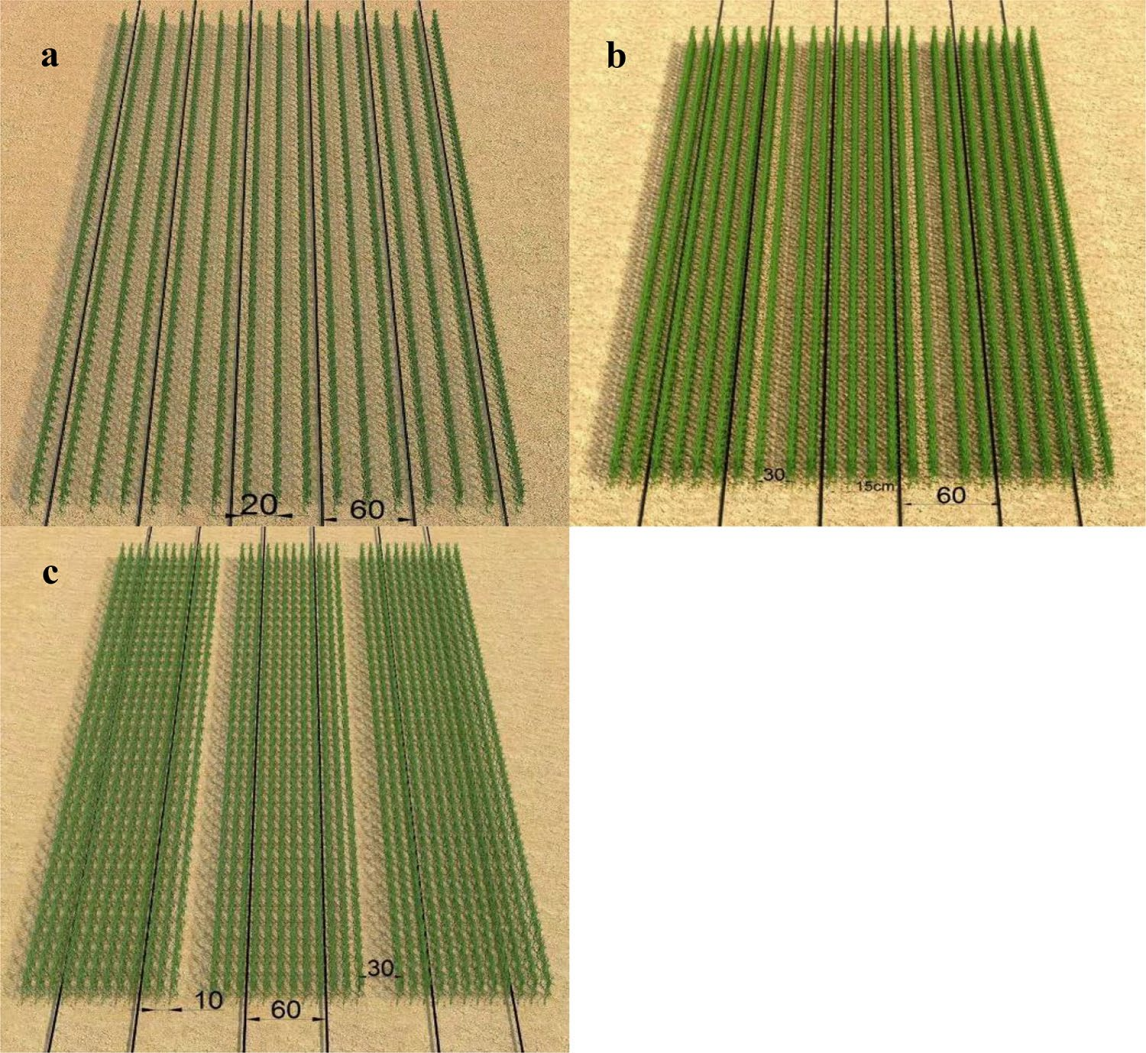
Planting patterns and drip tapes spacing.

This study was conducted using split plot experiment in a randomized complete block design with three replications. Planting patterns were as the main plots including three treatments as flows:

1. PA, conventional cropping with 20 cm equal row spacing and 60 cm spacing between drip tapes (Fig. 2a).
2. PB, strip cropping with the 15 cm row spacing, including 8 rows and 2 drip tapes with 60 cm spacing in each planting belt. There was 30 cm between each planting belt. (Fig. 2b).
3. PC, strip cropping with the 10 cm row spacing, including 12 rows and 2 drip tapes with 60 cm spacing in each planting belt. There was 30 cm between each planting belt. (Fig. 2c).

The subplots consisted of two super absorbent polymers (potassium polyacrylate-PAA) rates were designed as using (Y, 22.5 kg ha^−1^) and zero (N). Super absorbent polymers were applied annually as a single treatment and were broadcast with fertilizer prior to seeding and incorporated into the soil by cultivating, the depth of application was 20-25 cm.

The cropping layout of various drip irrigation treatments are illustrated in Fig. 2. Before sowing, total 18 plots (3 planting patterns × 2 water-retaining agent levels × 3 replications = 18 plots) were established. Each plot was 10 m long and 7.2 m wide. And in each plot, there had different rows and drip tapes among three planting patterns, including there were 37 rows and 13 drip tapes of PA, 40 rows and 10 drip tapes of PB, 60 rows and 10 drip tapes of PC. The Bayou No.1 oat cultivar was used for the experiments. The number of seeds used in all treatments was 312 per square meter. Crop planting was carried out via a hand push linear seeder and based on the designated cropping layouts. The soil tillage and sowing time, fertilizer amount and field management measures were the same for each treatment. The tillage system was spring cultivate. Compound granular fertilizer (15–15-15) was applied each year at 150 kg ha^−1^ resulting in 22.5 kg ha^−1^ nitrogen, 22.5 kg ha^−1^ phosphorous and 22.5 kg ha^−1^ potassium. All experimental plots were sown on 8th May in 2019 and on 12th May in 2020, and they were harvested on 27th August in 2019 and on 30th August in 2020 respectively.

The oat was treated with the supplemental irrigation (SI) management^38^ during the whole growth period. The time of irrigation included four stage: after sowing, tillering, jointing and anthesis. The amount of irrigation was dependent on the targeted soil relative water content and the plan wetting layer depth. In the study, the targeted soil relative water content was set to 70%, with a 20-cm-thick plan wetting layer. The soil water content in the plan wetting layers (*θ*_*m*_) and field capacity in the plan wetting layers (*FC*_*m*_) were determined at sowing, tillering, jointing and anthesis. And the targeted soil relative water content (*θ*_*r*_) was calculated by *θ*_*r*_ = *θ*_*m*_ ÷ *FC*_*m*_ × 100%. Water management occurred as follows: no irrigation when the *θ*_*r*_ was above 70%; when it was below 70%, irrigation was involved, and irrigation rate was calculated by *I*_*s*_ = 0.1 × *D*_*h*_ × *ρb* × (*FC*_*m*_ -*θ*_*m*_), where Dh (cm) was the plan wetting layer depth, ρb (g cm^−3^) was the soil bulk density in the plan wetting layer. The irrigation scheduling, effective precipitation and total irrigation values are given in Table 2 for different irrigation methods. An accurate flow meter was used to control the pre-determined irrigation amount.

**Table 2:** Irrigation volumes of different treatments (mm).

Year	Treatment		Irrigation time				Total
	Planting pattern	Super absorbent polymer	Sowing	Tillering	Jointing	Anthesis	
2019	PA	N	19.1	41.8	44.3	24.9	130.1
		Y	19.1	39.5	37.7	24.4	120.6
	PB	N	19.1	41.1	32.5	26.3	119.0
		Y	19.1	35.5	31.5	25.7	111.7
	PC	N	19.1	41.1	34.0	28.4	122.6
		Y	19.1	39.1	32.4	24.9	115.5
2020	PA	N	28.4	49.1	35.0	0	112.5
		Y	28.4	44.5	33.9	0	106.7
	PB	N	28.4	45.7	31.6	0	105.8
		Y	28.4	42.7	31.2	0	102.3
	PC	N	28.4	46.4	33.2	0	108.0
		Y	28.4	43.2	33.3	0	104.9

The discharge rate for drip tape is 2 L h^−1^. And each plot was independently equipped with a water meter to control the irrigation water volume. When the irrigation water volume reaches the demand, it will be closed. The pH of irrigation water is 8.35 and the EC of irrigation water is 514 µs/cm.

### Sampling and measurement methods

#### Soil water content and storage

Soil gravimetric water content (GWC, %) of 0–20, 20–40, 40–60,60–80, 80–100 cm soil layer at the different growth stages was determined by using the oven method at 105 ℃^39^. A diameter handheld soil auger was used to take the random soil samples in the middle part between the two rows of plants in each plots of the six treatments. And 5 samples were collected based on the method of “S” shape, and each sample was not in the same two rows. Besides, undisturbed soil core (100 cm^3^) from soil depth of 0–20, 20–40, 40–60,60–80, 80–100 cm was collected to determine soil bulk density^40^. Therefore, volumetric soil water storage (SWS, mm) was calculated by^41^ the following equation:

$$ SWS \, = \, GWC \, \times \, \rho b \, \times \, SD $$

where GWC is soil gravimetric water content, ρb (g cm^−3^) is soil bulk density and SD (mm) is referred to as the given soil depth.

#### Evapotranspiration.

Evapotranspiration was determined by the total precipitation, soil water storage consumption and irrigation because there was no surface runoff or groundwater during the growth period. The evapotranspiration were calculated by the following equations^41^:

$$ ET \, = \, P + \Delta W + I $$

where P is the total precipitation during the growth period, the ΔW is the change of soil water storage in the 0–100 cm soil layer and I is the irrigation volume during the growth period.

#### Yield and its components

Whole plots were harvested at the maturity stage each year to determine above-ground biomass and grain yield. Spike number of unit area was surveyed in the field, kernel number per spike and thousand-grain weight was tested in the science lab.

#### Water use efficiency

The water use efficiency (WUE, kg ha^−1^ mm^−1^) for grain yield were computed using the following equation^42^:

$$ WUE \, = \, Y/ET $$

where Y is grain yield, ET is evapotranspiration during the whole growth period of oat.

### Economic benefits

The conventional inputs were total labor and cost of seeds, fertilizer, water absorbing amendments, drip belts and agrochemicals. Total labor input used for field work consisted of plot preparation, field managements, sowing and harvesting, in the study, the total labor was the same among six treatments. The cost of drip belts only calculates the capillary cost between different treatments. The price of oat straw is calculated as 0.5 yuan kg^−1^, and the price of grain is calculated as 3.0 yuan kg^−1^ in both two years.

### Statistical analyses

The data, figures and tables were processed with the software of Microsoft Excel 2019 and IBM SPSS Statistics 25.0. All the data were analyzed by using one way analysis of variance (ANOVA), and the differences of mean values among different treatments were compared by the least significant difference (LSD) test (P < 0.05).

## Results

### Applied irrigation water volume

The volumes of applied irrigation water in the different treatments for the experimental years are presented in Table 2. Whether or not super absorbent polymers were applied, the irrigation volume all showed PA > PC > PB, the two strip cropping patterns both can reduce irrigation volume, respectively. Compared to PA, strip cropping patterns PB reduced applied irrigation water by 6.7–18.4 mm, and PC reduced by 4.5–14.6 mm. On the other hand, super absorbent polymers played an important role in affecting irrigation water. Compared with N, the irrigation water volume of Y under PA, PB and PC reduced by 7.65, 8.90 and 6.10 mm on average in two years, respectively.

### Soil water storage in the 0–100 cm soil layers

The ANOVA for soil water storage in the 0–100 cm soil layers is shown in Table 3. Growth periods (G), planting patterns (P) and super absorbent polymers (S) all had significant (P < 0.01) effect on soil water storage in the 0-100 cm soil layers, the interaction of super absorbent polymers (S) and growth periods (G) had significant (P < 0.01 or P < 0.05) effects and other interactions had no significant effects.

**Table 3:** ANOVA of effect of planting patterns, super absorbent polymers and growth period on soil water storage in 2019–2020.

Year	Factor						
	G	P	S	G*P	G*S	P*S	G*P*S
2019	**	**	**	NS	**	NS	NS
2020	**	**	**	NS	*	NS	NS

**Significant at 0.01 level, * significant at 0.05 level, NS means no significant. G, P and S represent growth stage, planting pattern and super absorbent polymer.

**Figure 3:**
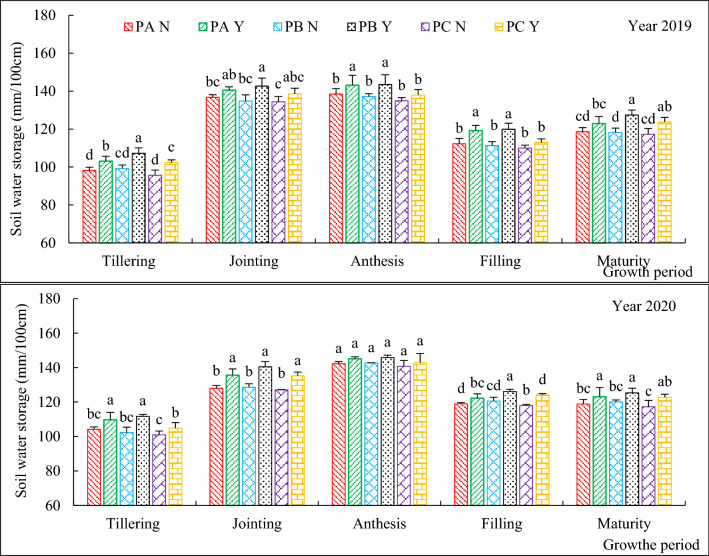
Soil water storage in the 0–100 cm soil layer as affected by planting patterns and super absorbent polymers in 2019–2020. *Notes* Smaller bar represents the standard error of mean (n = 3); Different lowercase letters stand for significance at 0.05 levels.

The soil water storage in the 0–100 cm soil layers for different treatments, at different growth period and in different years are shown in Fig. 3. Soil water storage of six treatments showed a same trend with the advance of growth period. When super absorbent polymers were not applied, three planting patterns had no significant effects on soil water storage in the whole growth period of oat. When super absorbent polymers were applied, three planting patterns showed different effects on soil water storage during different growth period, and the overall showed PB > PA > PC, but there showed inconsistent significant difference among three planting patterns. On the other hand, super absorbent polymers played the more important role in affecting soil water storage.

Under strip cropping with the 15 cm row spacing, the soil water storage of PBY was significant higher than that of PBN during the growing period of oat in two years except at anthesis in 2020, the improvement was observed up to 2.15%-9.23%. Under conventional cropping with 20 cm equal row spacing, the soil water storage of PAY was significant higher than that of PAN during the growth period of oat in two years except at jointing, maturity in 2019 and anthesis in 2020, the improvement was observed up to 1.99%-6.18%. Under strip cropping with the 10 cm row spacing, the soil water storage of PCY was significant higher than that of PCN at both tillering, maturity in 2019 and 2020, the other growth period showed different in two years, the improvement was observed up to 1.41%-7.16%.

### Grain yield and above-ground biomass

The ANOVA for grain yield and above-ground biomass are shown in Table 4. Planting pattern (P) and super absorbent polymers (S) and their interactions had significant (P < 0.05) effect on grain yield and above-ground biomass.

**Table 4:** ANOVA of effects of planting patterns, super absorbent polymers on grain yield and above-ground biomass of oat in 2019–2020.

Year	Factor	Grain yield	Above-ground biomass
2019	P	**	**
	S	**	**
	P*S	*	**
2020	P	**	**
	S	**	**
	P*S	**	**

**Significant at 0.01 level, * significant at 0.05 level. P and S represent planting pattern and water absorbing amendment.

**Figure 4:**
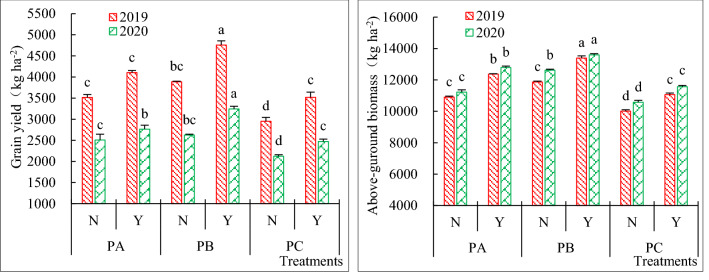
Grain yield and above-ground biomass of oat as affected by planting patterns and super absorbent polymers in 2019–2020. *Notes* Smaller bar represents the standard error of mean (n = 3); Different letters in the same year represent significant differences at 0.05 levels.

The planting patterns and water absorbing amendment effects on oat grain yield and above-ground biomass are presented in Fig. 4. When super absorbent polymers were applied, the oat grain yield and above-ground biomass of PBY were significant higher than that of PAY, the grain yield and above-ground biomass improvement were observed up to 16.65% and 7.31% on average in two years, respectively; the oat grain yield and above-ground biomass of PCY were significant lower than that of PAY, the grain yield and above-ground biomass reduction were observed up to 12.39% and 10.02% on average in two years, respectively. When super absorbent polymers were not applied, the oat grain yield and above-ground biomass of PBN and PAN were significantly higher than that of PCN, but there was no significant difference between PAN and PBN except the above-ground biomass in 2019.

Under the same planting pattern, the application of super absorbent polymers had the significant effects on oat grain yield and above-ground biomass. Compared with no application, the grain yield of PAY, PBY and PCY increased by 13.45%, 22.99% and 17.87% on average in two years, respectively. And the corresponding above-ground biomass increased by 13.64%, 10.28% and 9.99%.

### Yield components

The ANOVA for yield components in Table 5. Planting pattern (P) and super absorbent polymers (S) had significant (P < 0.05 or P < 0.01) effect on spike number, kernel number per spike, thousand grain weight, spike length and grain weight per spike. The interactions of P and W had significant effect on spike number (P < 0.01) and kernel number per spike (P < 0.05).

**Table 5:** Yield components as affected by planting patterns and super absorbent polymers in 2019–2020.

Year	Treatment		Spike number (plants m^−2^)	Kernel number per spike	Thousand grain weight (g)	Spike length (cm)	Grain weight per spike (g)
	Planting pattern	Super absorbent polymer					
2019	PA	N	642.38 ± 7.64c	53.00 ± 2.52bc	22.88 ± 0.99bc	15.33 ± 0.65bc	1.45 ± 0.11b
		Y	668.32 ± 13.97b	55.33 ± 2.52b	23.91 ± 0.79ab	15.57 ± 0.50bc	1.58 ± 0.09ab
	PB	N	662.77 ± 10.48b	57.00 ± 1.73b	23.38 ± 0.10ab	15.97 ± 0.59ab	1.57 ± 0.11a
		Y	688.91 ± 10.80a	63.67 ± 3.06a	24.05 ± 0.28a	16.83 ± 0.61a	1.69 ± 0.06a
	PC	N	600.15 ± 9.18d	46.33 ± 2.52d	22.54 ± 0.17c	14.99 ± 0.51c	1.27 ± 0.10c
		Y	636.53 ± 11.19c	50.67 ± 2.08c	22.61 ± 0.04c	15.27 ± 0.23c	1.37 ± 0.13c
ANOVA results		P	**	**	*	**	**
		S	**	**	*	*	*
		P*S	**	*	NS	NS	NS
2020	PA	N	616.59 ± 8.49c	47.67 ± 1.53b	22.01 ± 0.07bc	14.14 ± 0.53bc	1.14 ± 0.08bc
		Y	645.48 ± 9.27b	50.33 ± 1.53b	22.79 ± 0.61ab	14.88 ± 0.85ab	1.27 ± 0.04ab
	PB	N	641.28 ± 4.91b	49.67 ± 1.53b	22.28 ± 0.28abc	14.89 ± 0.62ab	1.21 ± 0.02b
		Y	671.91 ± 2.17a	55.67 ± 4.04a	23.10 ± 0.69a	15.85 ± 0.20a	1.39 ± 0.15a
	PC	N	595.15 ± 12.00d	40.33 ± 1.53c	21.37 ± 0.58c	13.54 ± 0.67c	1.04 ± 0.06c
		Y	621.89 ± 12.32c	45.33 ± 4.51bc	21.72 ± 0.34c	14.22 ± 0.35bc	1.13 ± 0.08b
ANOVA results		P	**	**	*	**	**
		S	**	**	*	*	**
		P*S	**	*	NS	NS	NS

Different letters of different treatments in the same year showed significant differences (P < 0.05), and the following numbers showed the standard deviation of the data. P and S represent planting pattern and super absorbent polymer.** significant at 0.01 level, * significant at 0.05 level, NS means no significant.

The planting patterns and super absorbent polymers effects on oat grain components in Table 5. When super absorbent polymers were applied, compared with PAY, PBY significantly increased the spike number and kernel number per spike of in both two years and there had no significant effects on the thousand grain weight, grain weight per spike and spike length between PBY and PAY except spike length in 2019; PCY significantly decreased the spike number and thousand grain weight in both two years and there had no significant effects on the grain weight per spike and spike length between PCY and PAY. When super absorbent polymers were not applied, compared PAN, PBN significantly increased the spike number in both two years and had no significant effects on other yield components except grain weight per spike in 2019; PCN significantly decreased the spike number and kernel number per spike in both two years.

Under the same planting pattern, the application of super absorbent polymers had significant effects on spike number in three planting patterns in both two years. However, application of super absorbent polymers only significantly increased the kernel number per spike in strip cropping with the 15 cm row spacing in two years. And there all had no significant effects on thousand grain weight, spike length and grain weight per spike between application of water absorbing amendment and not application in three planting patterns.

### Evapotranspiration and its proportion

The planting pattern and super absorbent polymers effects on evapotranspiration and its proportion in Table 6. Compared with PA, whether or not super absorbent polymers were applied, PB both significantly decreased the total evapotranspiration of oat by 2.55% (PBY) and 2.08% (PBN) on average in two years; PC significantly decreased the total evapotranspiration in 2019.

**Table 6:** Evapotranspiration and its proportion as affected by planting patterns and super absorbent polymers in 2019–2020.

Year	Treatment		Total evapotranspiration	Proportion					
	Planting pattern	Water absorbing amendments		Soil water storage consumption		Precipitation		Irrigation	
				Quantity (mm)	Ratio (%)	Quantity (mm)	Ratio (%)	Quantity (mm)	Ratio (%)
2019	PA	N	426.93 ± 4.17a	11.00 ± 1.88a	2.58 ± 0.43b	285.8	66.94 ± 0.29e	130.1	30.48 ± 0.13a
		Y	413.17 ± 3.60c	6.79 ± 1.50b	1.64 ± 0.36c	285.8	69.17 ± 0.25c	120.6	29.18 ± 0.11b
	PB	N	418.27 ± 1.21bc	13.46 ± 1.21a	3.22 ± 0.28a	285.8	68.33 ± 0.20d	119.0	28.45 ± 0.08c
		Y	399.70 ± 3.01e	2.16 ± 0.82c	0.54 ± 0.20d	285.8	71.50 ± 0.15a	111.7	27.95 ± 0.06d
	PC	N	420.85 ± 3.89b	12.40 ± 1.64a	2.95 ± 0.38b	285.8	67.91 ± 0.27f.	122.6	29.14 ± 0.11b
		Y	407.16 ± 3.21d	5.89 ± 1.00b	1.45 ± 0.24c	285.8	70.19 ± 0.17b	115.5	28.35 ± 0.07c
2020	PA	N	373.07 ± 3.08a	7.69 ± 1.60a	2.06 ± 0.42a	252.9	67.79 ± 0.29d	112.5	30.15 ± 0.13a
		Y	363.03 ± 0.73b	3.38 ± 0.73bc	0.93 ± 0.20bc	252.9	69.66 ± 0.14bc	106.7	29.40 ± 0.06b
	PB	N	365.13 ± 2.45b	6.45 ± 2.45ab	1.76 ± 0.66ab	252.9	69.26 ± 0.47c	105.8	28.97 ± 0.20c
		Y	356.34 ± 0.71c	1.19 ± 0.33c	0.33 ± 0.09c	252.9	70.97 ± 0.07a	102.3	28.69 ± 0.03d
	PC	N	370.23 ± 3.27a	9.30 ± 3.00a	2.51 ± 0.79a	252.9	68.31 ± 0.55d	108.0	29.18 ± 0.24bc
		Y	361.53 ± 2.01b	3.74 ± 1.07bc	1.03 ± 0.29bc	252.9	69.95 ± 0.21b	104.9	29.01 ± 0.09c

Different letters of different treatments in the same year showed significant differences (P < 0.05), and the following numbers showed the standard deviation of the data.

The evapotranspiration of oat still mainly depended on the natural precipitation, and the secondly depended on irrigation (Table 6). When super absorbent polymers were applied, compared with PAY, PBY significantly increased the precipitation ratio by 2.64%, decreased the soil water storage consumption quantity and its ration by 66.46% and 65.59% and decreased irrigation ration by 3.32% on average in two years; PCY only significantly increased the precipitation ratio in 2019 and decreased the irrigation ration by 2.08% on average in two years. When super absorbent polymers were not applied, there had not significant effects on soil water storage consumption quantity among three planting patterns. Compared with PAN, PBN significantly increased precipitation ratio by 2.13% and decreased irrigation ration by 5.28% on average in two years; PCN significantly increased precipitation ratio by 1.45% in 2019 and decreased irrigation ration by 3.81% on average in two years.

Under the same planting pattern, the application of super absorbent polymers all had the significant effects on soil water storage consumption quantity and its ration, precipitation ratio and irrigation ration except PC in 2020. Compared with PAN, PBN and PCN, PAY, PBY and PCY increased the precipitation ratio by 3.05%, 3.56% and 2.89%; decreased the soil water storage consumption quantity and its ration by 37.27% and 55.38%, 83.57% and 81.30%, 52.70% and 59.26%; decreased the irrigation ration by 3.36%, 1.35% and 1.63%.

### Water use efficiency

The planting pattern and super absorbent polymers effects on water use efficiency in Table 7. Compared with PA, whether or not super absorbent polymers were applied, PB both significantly increased the WUE by 19.70% (PBY) and 9.87% (PBN) on average in two years; PC both significantly decreased the WUE by 11.57% (PCY) and 14.79% (PCN) on average in two years. And under the same planting pattern, the application of super absorbent polymers all had the significant effects on WUE, respectively.

**Table 7:** Water use efficiency as affected by planting patterns and super absorbent polymers in 2019–2020.

Year	Treatment		WUE
	Planting patterns	Super absorbent polymers	
2019	PA	N	8.23 ± 0.20e
		Y	9.93 ± 0.10b
	PB	N	9.29 ± 0.02c
		Y	11.90 ± 0.22a
	PC	N	7.01 ± 0.21f.
		Y	8.64 ± 0.32d
2020	PA	N	6.72 ± 0.38d
		Y	7.61 ± 0.26b
	PB	N	7.19 ± 0.05c
		Y	9.10 ± 0.17a
	PC	N	5.73 ± 0.14e
		Y	6.84 ± 0.14 cd

Different letters of different treatments in the same year showed significant differences (P < 0.05), and the following numbers showed the standard deviation of the data.

### Economic benefits

The planting pattern and super absorbent polymers effects on economic benefits in Table 8. Whether or not super absorbent polymers were applied, the economic benefits both showed PB > PA > PC. Compared with PA, two strip cropping patterns reduced the costs as a result of a decrease in the number of drip tape tubes. PB increased economic benefits by 3509.64 yuan ha^−1^ (PBY) and 2435.81 yuan ha^−1^ (PBN) on average in two years, respectively. So strip cropping with the 15 cm row spacing (PB) had the better production benefits in drip irrigation planting of oat, although application of super absorbent polymers increased the cost, but the improvement of economic benefits was higher.

**Table 8:** Economic benefits as affected by planting patterns and super absorbent polymers in 2019–2020.

Year	Treatment		Output (yuan ha^−1^)		Cost (yuan ha^−1^)		Economic benefits (yuan ha^−1^)
	Planting pattern	Super absorbent polymers	Grain	Straw	Conventional cost	Drip belts cost	
2019	PA	N	10,545.13	5451.95	3750.00	7500.00	4747.08
		Y	12,308.23	6183.75	4050.00	7500.00	6941.98
	PB	N	11,662.35	5934.45	3750.00	6150.00	7696.79
		Y	14,274.69	6696.56	4050.00	6150.00	10,771.24
	PC	N	8852.83	5002.96	3750.00	6150.00	3955.80
		Y	10,552.50	5532.39	4050.00	6150.00	5884.89
2020	PA	N	7525.38	5614.19	3750.00	7500.00	1889.57
		Y	8291.46	6392.83	4050.00	7500.00	3134.29
	PB	N	7870.90	6310.56	3750.00	6150.00	4281.46
		Y	9727.08	6797.22	4050.00	6150.00	6324.31
	PC	N	6367.07	5287.83	3750.00	6150.00	1754.90
		Y	7420.04	5784.84	4050.00	6150.00	3004.88

## Discussion

### Effect of planting patterns and super absorbent polymer on soil water storage

Row spacing can affect soil water storage by affecting the distribution and growth of roots in the soil, and crop root system is the key factor to change the physical structure of soil^43,44^. Through the growth of crop roots in the soil, a series of biological pores are formed in the soil, so as to improve the water conductivity of the soil^45,46^.Our data showed that the change of single row spacing did not exhibit any obvious effects on soil water storage during the course of this study (Fig. 3). The differences in soil water storage over the growth period were caused by irrigation and rainfall. And super absorbent polymers played the more important role in affecting soil water storage. This was consistent with Farrell^47^ who reported that super absorbent polymers improved water holding capacity. And super absorbent polymers retained the rainfall and irrigation, lowered evaporation losses and increased plant available water for crop growth^48,49^. After the application of super absorbent polymers, it can improve the water holding characteristics of soil, improve the water supply capacity of soil, lock the water in soil, form a "micro reservoir" in the rhizosphere of crops, and slowly release it for the needs of crop growth. This is mainly due to the structure of the super absorbent polymers, which contain hydrophilic functional groups. After being applied to the soil, it can adsorb the inorganic ions around the soil, and stimulate the impact on the soil structure under the effect of soil water regulation. It can increase the soil porosity, promote the spreading performance of molecules in the soil, improve the soil structure, and further promote the improvement of soil water storage capacity. Our results showed that application of super absorbent polymers in three planting patterns all can increased soil water storge, the improvement was up to 1.41%-9.23% (Fig. 3) and this effects showed more better in striping cropping with 15 cm row spacing and effects of combination of planting patterns with super absorbent polymers had showed the obviously after anthesis of oat.

### Effect of planting patterns and super absorbent polymer on yield and its components

Oat yield is composed of spike number, kernel number per spike, thousand grain weight. Under different planting modes, each yield component factor changes according to the influence of the environment and the characteristics of the crop itself. The yield components are affected by environmental factors such as water conditions and temperature^50^. Different cultivation measures which including sowing date^51^, planting density^52^, row spacing^53,54^, fertilizer^51^, irrigation^34^ can have significant effects on oat yield. In our study, under three planting patterns, application of super absorbent polymers all had the significant (P < 0.05) effects on grain yield and above-ground biomass (Fig. 4), and also had the significant (P < 0.05) effects on spike number and kernel number per spike. This was consistent with Wu^35^ who reported that super absorbent polymers can increase oat yield under drip irrigation. And this study showed that the key reason for improving oat grain yield under drip irrigation lies in the changes of spike number and thousand grain weight which was different with our study. Our data showed whether or not super absorbent polymers were applied, strip cropping with 10 cm row spacing both significantly decreased the grain yield and above-ground biomass (Fig. 4), which may be caused by that excessive decreased row spacing drastically decreases the light transmission ratio, thus decreasing the lighting conditions of the canopy. This was basically consistent with the previous research results on the effects of excessively narrowing row spacing on wheat yield^55^.Compared with conventional cropping with 20 cm equal row spacing, strip cropping with 15 cm row spacing significantly (P < 0.05) increased the grain yield, kernel number and spike number when super absorbent polymers were applied. But when the super absorbent polymers were not applied, the effects were not significant, the reasons maybe although the row spacing can improve the population distribution of oats and have the potential of increasing production, these were not significant in the superior growth environment. It can be seen that in the drip irrigation cultivation of oats, the best effect can be achieved by considering the joint action of multiple factors.

### Effect of planting patterns and super absorbent polymer on water use of oat

The studies showed that reducing row spacing can significantly inhibit ineffective evaporation when soil water can meet the needs of crop growth. Under the same planting density, the reduction of wheat planting row spacing to 7.5 cm can effectively reduce the ineffective water consumption during the growth period of winter wheat and improve its water use efficiency. Sun^56^ and Li^57^ studied that wheat planting with 15 cm row spacing had the moderate total water consumption and could effectively improve water use efficiency. And a study also showed that under the row spacing of 7.5 cm, 15 cm and 30 cm, the water consumption of winter wheat increased and the water use efficiency decreased with the increasing of row spacing^58^. There were many studies about effects of super absorbent polymers on soil water consumption and water use efficiency in recent years. And most of these studies all showed that super absorbent polymers had effects of reducing soil water consumption and improving the utilization efficiency of rainfall and irrigation water^59,60^. Yang^60^ studied that application of super absorbent polymers could effectively reduce the water consumption during the whole growth period of maize and under the condition that the rainfall was higher than the water consumption, the excess rainfall could be effectively stored in the soil. And the reasons was that the super absorbent polymers can significantly prolong the stable evaporation period of soil moisture, thus prolonging the evaporation time of soil moisture and reducing the consumption of soil moisture. In our study, compared with conventional cropping with 20 cm equal row spacing, the two strip cropping planting patterns both reduced the irrigation volume (Table 2) and the strip cropping with 15 cm row spacing had the lowest irrigation volume (Table 2). Our data showed under three planting patterns, the application of super absorbent polymers all had the effects on reducing the total evapotranspiration (Table 6), which were different with the results of Du^61^ and Tian^62^ who considered that the application of super absorbent polymers had no significant effects on the total evapotranspiration. These maybe because that our study had the different planting environment with the two studies which the experiments were conducted in dry farm land and the application of super absorbent polymers in our study also improved the distribution of water consumption from different sources during the growth period of oats. Weather or not the super absorbent polymers were applied, compared with the conventional cropping with 20 cm equal row spacing, strip cropping with 15 cm row spacing both significantly (P < 0.05) reduced the total evapotranspiration (Table 6). Our results indicate that, although the two strip both reduced the total evapotranspiration and irrigation volume, but the water use efficiency showed different between two strip cropping patterns, while water use efficiency is determined by yield and water consumption. Compared with the conventional cropping with 20 cm equal row spacing strip cropping with 15 cm row spacing significantly (P < 0.05) increased the water use efficiency, although strip cropping with 10 cm row spacing significantly (P < 0.05) decreased.

## Conclusion

The results of the present study indicate that when implementing drip irrigation for oat crops, the selecting of an appropriate oat row spacing layout between drip irrigation tapes, establishing planting belt around drip irrigation tapes and applying super absorbent polymers will increase the yield and water use efficiency, while reducing the costs as a result of a decrease in the number of drip tape tubes. Thus, a drip irrigation oat planting pattern was proposed, which reduced the row spacing of oat planting from 20 to 15 cm, adjusted the equal row spacing planting to 8-row strip planting, with a belt spacing of 30 cm, combined with the application of 22.5 kg ha^−2^ applying super absorbent polymers. And this oat planting pattern is a viable strategy to improve oat productivity. This study evaluated the proposed planting methods from the perspective of oat yield and water use efficiency. In the future, the amount and time of oat irrigation should be further optimized in the proposed planting methods in order to further save water and achieve high efficiency.

## Supplementary Information

Supplementary Information.

## Data Availability

All data generated or analysed during this study are included in this published article and its supplementary information files.
